# Modulation of Phagosomal pH by *Candida albicans* Promotes Hyphal Morphogenesis and Requires Stp2p, a Regulator of Amino Acid Transport

**DOI:** 10.1371/journal.ppat.1003995

**Published:** 2014-03-13

**Authors:** Slavena Vylkova, Michael C. Lorenz

**Affiliations:** Department of Microbiology and Molecular Genetics, The University of Texas Health Science Center, Houston, Texas, United States of America; University of Rochester, United States of America

## Abstract

*Candida albicans*, the most important fungal pathogen of humans, has a unique interaction with macrophages in which phagocytosis induces a switch from the yeast to hyphal form, allowing it to escape by rupturing the immune cell. While a variety of factors induce this switch in vitro, including neutral pH, it is not clear what triggers morphogenesis within the macrophage where the acidic environment should inhibit this transition. In vitro, *C. albicans* grown in similar conditions in which amino acids are the primary carbon source generate large quantities of ammonia to raise the extracellular pH and induce the hyphal switch. We show here that *C. albicans* cells neutralize the macrophage phagosome and that neutral pH is a key inducer of germination in phagocytosed cells by using a mutant lacking *STP2*, a transcription factor that regulates the expression of multiple amino acid permeases, that is completely deficient in alkalinization *in vitro*. Phagocytosed *stp2Δ* mutant cells showed significant reduction in hypha formation and escaped from macrophages less readily compared to wild type cells; as a result *stp2Δ* mutant cells were killed at a higher rate and caused less damage to RAW264.7 macrophages. Stp2p-regulated import leads to alkalinization of the phagosome, since the majority of the wild type cells fail to co-localize with acidophilic dyes, whereas the *stp2Δ* mutant cells were located in acidic phagosomes. Furthermore, *stp2Δ* mutant cells were able to form hyphae and escape from neutral phagosomes, indicating that the survival defect in these cells was pH dependent. Finally, these defects are reflected in an attenuation of virulence in a mouse model of disseminated candidiasis. Altogether our results suggest that *C. albicans* utilizes amino acids to promote neutralization of the phagosomal pH, hyphal morphogenesis, and escape from macrophages.

## Author Summary

The innate immune system represents a key barrier that fungal pathogens such as *Candida albicans* must overcome in order to disseminate through the host. *C. albicans* cells phagocytosed by macrophages initiate a complex program that involves a large-scale reprogramming of metabolism and transcription and results in the switch to a hyphal form that can penetrate and kill the macrophage. Though a number of signals are known to induce this morphological transition in vitro, what does so following phagocytosis has been unclear. We previously showed that *C. albicans* rapidly neutralizes acidic, nutrient-poor media that resembles the phagolysosome and that this is deficient in mutants impaired in amino acid import due to a mutation in *STP2*. In this paper, we investigate whether this happens within the macrophage as well. We show here that, in contrast to wild-type cells, *stp2Δ* mutants occupy an acidic phagosome and are unable to initiate hyphal differentiation. Because of this, they are more sensitive to killing and do less damage to the macrophages than cells that can neutralize the phagolysosome. We conclude that alteration of phagosomal pH is an important virulence adaptation in this species.

## Introduction

Normally a benign commensal, *Candida albicans* is also the most prevalent fungal pathogen in humans. Common mucosal manifestations of candidiasis are oropharyngeal thrush and vaginitis, but *C. albicans* can infect virtually any body site [1], [2]. The most serious infection – disseminated hematogenous candidiasis – is the fourth most common acquired hospital infection with a mortality rate of about 40% [3], [4]. In healthy individuals the innate immune system maintains *C. albicans* as a commensal and, with the exception of vaginitis, *C. albicans* infections are associated with defects in innate immunity. A variety of factors such as neutropenia, chemotherapy, implanted medical devices, and several genetic disorders have been linked with increased risk for disseminated candidiasis, emphasizing the important role of the immune system, including phagocytes such as macrophages and neutrophils [5].

Phagocytosis is an important step in the process by which macrophages destroy foreign cells. Many pathogens have evolved strategies to avoid or subvert phagocytosis at various stages of this process. For instance, bacterial pathogens such as *Pseudomonas aeruginosa* and *Yersinia enterolitica* inhibit phagocytosis through direct inhibition or by altering cell surface structure [6], [7]. Other pathogens, such as *Anaplasma phagocytophila*, interfere with the endocytic process or with the activity of macrophage-derived antimicrobial factors [8], [9]. *Mycobacterium tuberculosis*, *Salmonella typhimurium*, *Listeria monocytogenes* and other pathogens have developed strategies to either withstand or modulate the acidic pH of the phagolysosome, and/or alter fusion of the phagosome with the lysosome to prevent killing [10]–[12]. Similarly, *C. albicans* has developed strategies to escape phagocytosis and killing by the macrophages. Inside the macrophage *C. albicans* differentiates into the filamentous hyphal form, which ruptures the macrophage allowing it to escape and resume proliferation. This morphogenetic switch is required for virulence and has therefore been well studied [13], [14]. A variety of factors can trigger morphogenesis *in vitro*, including neutral pH, serum, elevated CO_2_ concentration, physiological temperature, and N-acetyl glucosamine [15]. While several of these inducing factors (37°C, elevated CO_2_) act on phagocytosed cells, an acidic phagosome should inhibit germination and it has been unclear what stimulates this morphological transition. Understanding this phenomenon is further complicated by evidence suggesting that *C. albicans* might modulate the phagosomal milieu or alter endocytic trafficking [16]. In fact, the exact nature of the intracellular compartment(s) containing *C. albicans* is not clear; here we use the generic term phagosome for simplicity.

The morphogenetic change is only part of the response to phagocytosis. Genomic and proteomic profiling indicates that *C. albicans* responds to phagocytosis by a significant reorganization of metabolic processes [17]–[20]. The response of *C. albicans* within macrophages is broadly similar to that seen after nutrient starvation, including repression of translation and glycolysis and activation of metabolic pathways required to use less favored carbon sources, including the glyoxylate cycle, β-oxidation, and gluconeogenesis [17], [18], [21]. Some of these metabolic pathways have been shown to be required for full virulence or are induced *in vivo* in animal models of candidiasis indicating that cells experience carbon starvation in the context of the intact host [19], [22]–[24]. Growth in alternative carbon sources also alters recognition by phagocytes, drug resistance and other relevant phenotypes [25], [26].

In a recent study we demonstrated that *C. albicans* cells grown in acidic medium with a nutrient composition similar to that predicted to exist inside phagocytic cells rapidly alkalinize the environment [27]. This phenomenon requires amino acid catabolism and results in extrusion of as ammonia presumably derived from the amino acid. As the cells neutralize the media, they auto-induce a shift to the hyphal form [27]. We identified mutations in several genes that impaired or abolished alkalinization, with the most severe phenotype conferred by mutation of *STP2*, which encodes for a transcription factor that regulates expression of amino acid permeases. We previously postulated that *C. albicans* might modulate the pH of the phagosome [27], a suggestion also made by others [16]. Thus, the *stp2Δ* mutant is an excellent tool with which to probe the importance of extracellular alkalinization during contact with host phagocytes.

Here we test the ability of *C. albicans* cells to alkalinize the phagosomal space and the importance of neutral pH in escape from phagocytosis. We show that phagocytosed *stp2Δ* mutant cells do not underdo hyphal morphogenesis within macrophages, suggesting that amino acid uptake is necessary for this process. Cells lacking *STP2* are defective in killing macrophages and show reduced survival upon phagocytosis. Finally, we show that wild type *C. albicans* cells can modulate the intraphagosomal pH, since these cells fail to co-localize with acidophilic dyes. In contrast, *stp2Δ* mutant cells inhabit acidic phagosomes, suggesting that the alteration of phagosomal pH by *C. albicans* requires amino acid catabolism, as does alkalinization in vitro. Pharmacological neutralization of the phagosome restored hyphal morphogenesis in the *stp2Δ* mutant cells, indicating that neutral pH is a critical inducing factor in this environment. Consistent with this, *stp2Δ* mutants were attenuated in virulence in a mouse model of disseminated candidiasis. Altogether our results suggest that during phagocytosis *C. albicans* utilizes amino acids to promote neutralization of the phagosome, stimulation of hyphal morphogenesis and escape from the macrophages and that this process contributes to fitness within the host.

## Results

### Construction and verification of new *stp2Δ* mutants

*C. albicans* cells grown in conditions that mimic the phagolysosome efficiently alkalinize the environment, resulting in induction of hyphal morphogenesis. We hypothesized that this phenomenon may occur within the phagosome to promote fungal survival and escape. To test this, we took advantage of cells carrying mutation in the *STP2* gene, a regulator of amino acid permease expression [28], since cells lacking *STP2* are defective in environmental alkalinization [27]. Because the existing *stp2Δ* mutant strain is apparently aneuploid [28] and uses *URA3* as a disruption marker, which can be problematic when used for *in vivo* and *ex vivo* studies [29], [30], we generated two independent *stp2Δ* mutant strains (SVC17, SVC18) using the SAT-flipper method [31] as described in the Materials and Methods.

**Figure 1:**
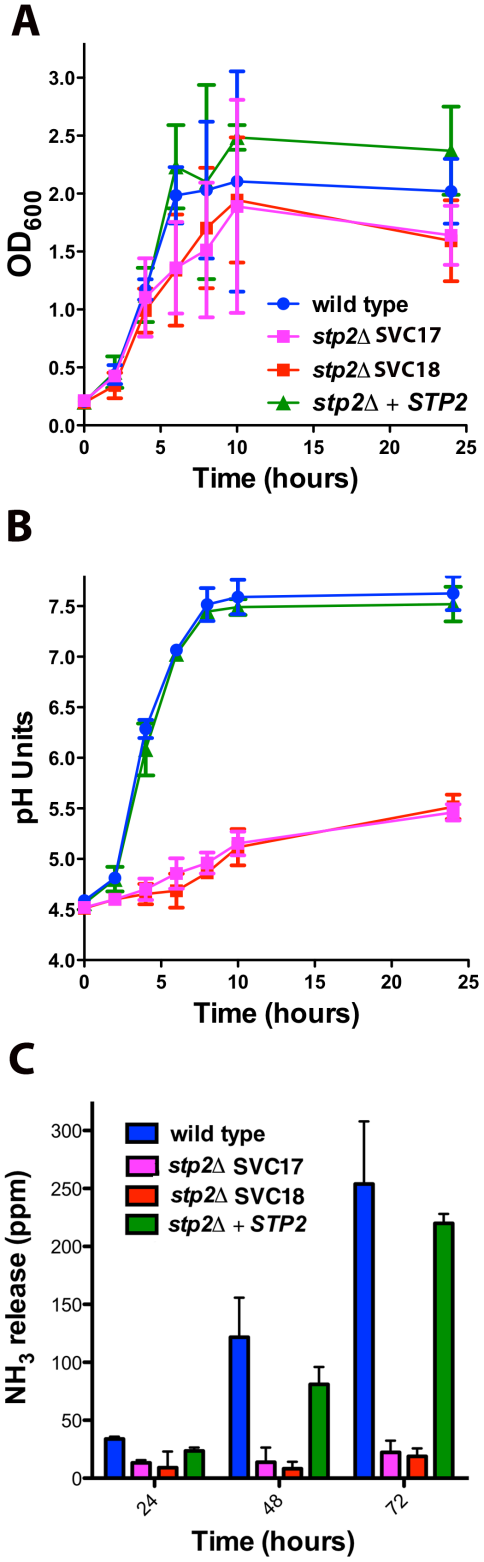
*C. albicans* s*tp2Δ* mutant cells fail to change the environmental pH. **A.** *C. albicans* strains of the indicated genotypes were grown in YNB+1% CAA, pH 4.5 at 37°C and growth was assessed by measuring OD_600_ at the indicated time points. **B.** The pH of the same cultures was measured. **C.** *C. albicans* strains were grown on GM-BCP agar to promote alkalinization and ammonia release was quantified as noted in Materials and Methods. Each experiment was performed in triplicate.

In order to confirm the phenotype in the newly generated strains we verified that they shared previously published phenotypes in amino acid assimilation, alkalinization and ammonia release [27], [28]. Our newly generated strains behaved identically to the previously published *stp2Δ* mutant strain PMRCA57 [28] when grown on multiple amino acids as the sole nitrogen source and phenotypes were fully complemented in the restored strain (Fig. S1). Next, we tested if the newly developed strains were able to alkalinize an acidic environment via production of ammonia. The *stp2Δ* mutant strains grew slightly more slowly than the wild type cells in alkalinization-inducing medium (YNB+1% CAA, pH 4.5), but the difference was not statistically significant (Fig. 1A). In contrast, the *stp2Δ* mutant cells showed a dramatic defect in alkalinization (Fig. 1B). In cultures with the wild type and complemented strains, the media pH rose from a starting pH of 4.5 to about 7.5 within seven hours, whereas cultures with the *stp2Δ* mutant reached only pH 5.4 at 24 hours (Fig. 1B). A similar environmental alkalinization defect was observed in the *stp2Δ* mutant when cells were grown in medium M199 (data not shown) and on the solid medium GM-BCP, pH 4.5 (Fig. S2). Alkalinization is associated with extrusion of volatile ammonia [27] and non-alkalinizing *stp2Δ* mutant colonies did not release significant quantities of ammonia when grown on GM-BCP medium (Fig. 1C). Overall, these results confirm that the newly generated *stp2Δ* mutant strain shows the expected phenotypes: impaired utilization of amino acids, a severe defect in the ability to modulate the environmental pH, and abrogated release of volatile ammonia during this process [27], [28].

**Figure 2:**
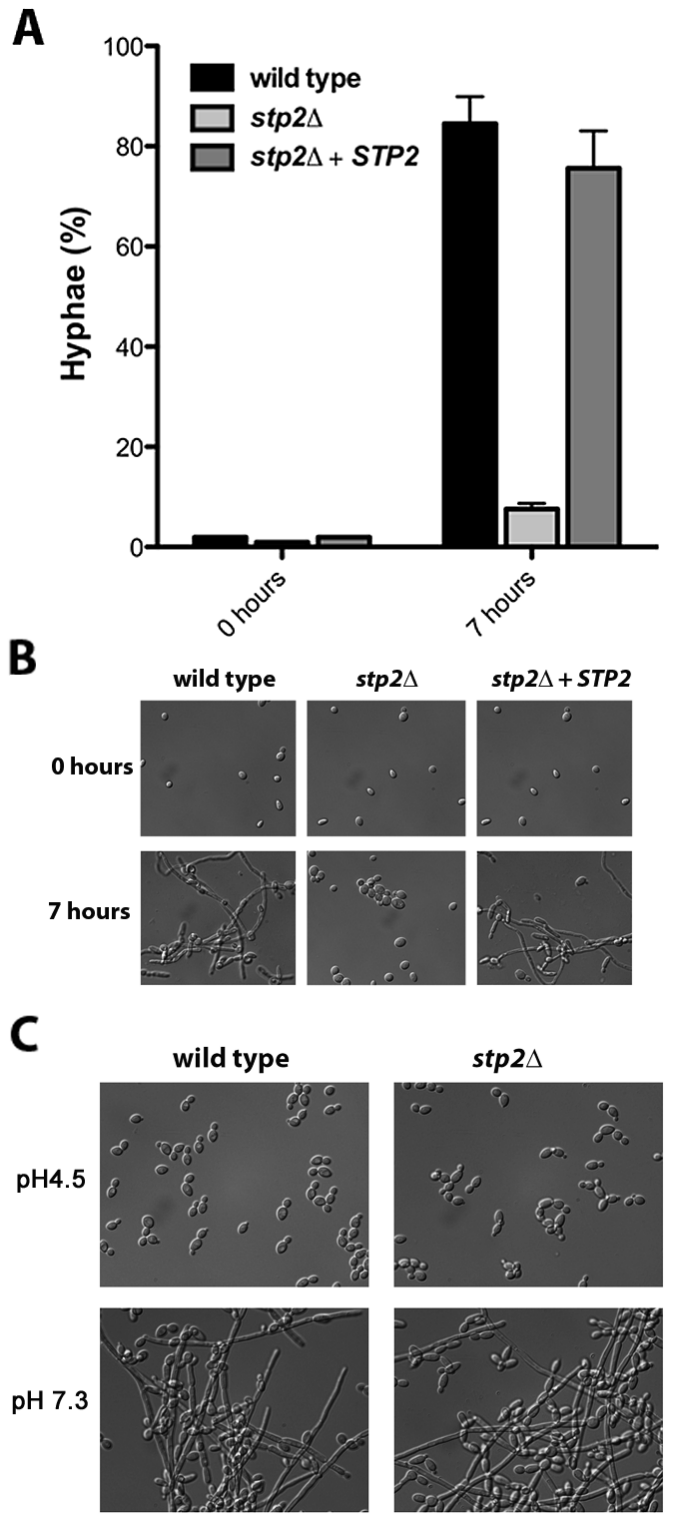
Cells lacking *STP2* do not induce hyphal morphogenesis under alkalinization conditions. **A.** Strains of the indicated genotype were grown overnight in YPD and diluted to OD600 ∼0.2 in YNB + 1% CAA, pH 4.5. Cellular morphology was assessed in photomicrographs of samples after seven hours by counting at least 100 cells per condition. **B.** Representative images from the experiment in (A) are shown. **C.** *C. albicans* wild type or *stp2Δ* strains were grown overnight in YPD, then transferred to YPD medium at pH 4.5 or 7.3. Cells were incubated at 37°C and after 24 hours samples were collected and photographed. The data in panels B and C are for a single experiment, but are representative of multiple replicates. The scale bars in panels B and C are 20 µm.

By generating a neutral pH, alkalinization induces *C. albicans* to switch from yeast to hyphal form, which is not observed when alkalinization is repressed by glucose or buffering [27]. Therefore, we assessed the morphology of the *stp2Δ* mutant cells grown under alkalinizing conditions (Fig. 2A). As expected, the majority of the wild type cells (∼85%) and of the *STP2* complemented cells (∼75%) switched to the hyphal morphology as the media neutralized. In contrast, fewer than 5% of the *stp2Δ* mutant cells had switched to the hyphal form within seven hours, at which point the pH of the medium was 4.8. Representative images of each strain prior to and after alkalinization of the medium are presented in Fig. 2B. To verify that the observed defect in morphological switch is due to the inability of the *stp2Δ* mutants to raise the environmental pH under these conditions and not due to an inherent defect in responding to neutral pH, we assessed hypha formation in the wild type and *stp2Δ* mutant cells in standard YPD medium at pH 4.5 or 7.3 (average pH achieved by wild-type cells). We did not observe any difference in the morphologies of the wild type and the *stp2Δ* mutant cells (Fig. 2C). Thus, the inability of the *stp2Δ* mutant cells to switch to the hyphal form in alkalinizing conditions is not due to a general morphological defect in these cells.

### *stp2Δ* mutant cells fail to escape phagocytosis

**Figure 3:**
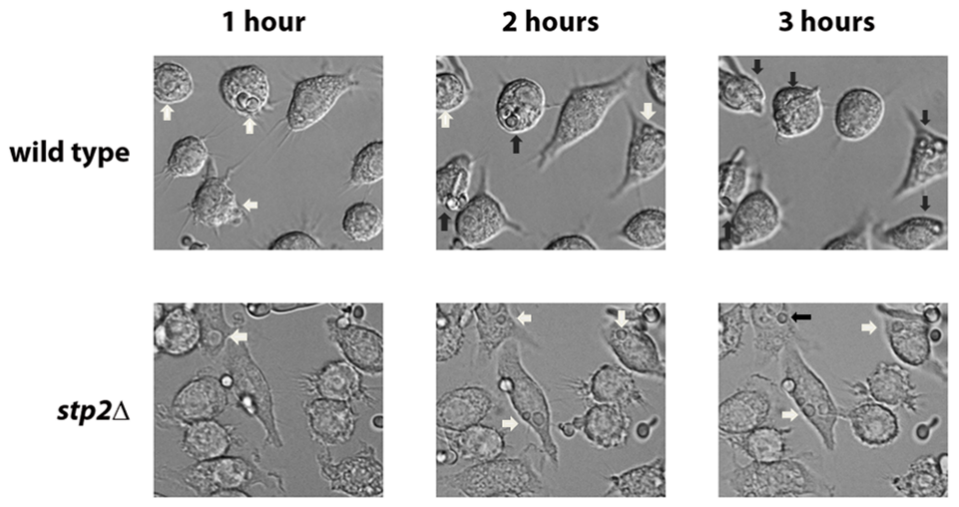
Cells lacking *STP2* fail to form hyphae and escape from phagocytosis. Log phase wild type cells or *stp2Δ* mutant cells were co-cultured with RAW264.7 macrophages in RPMI medium. The interaction was observed using time-lapse microscopy as noted in Materials and Methods. Representative images are shown. White arrows indicate cells in yeast morphology, whereas black arrows point to germinated cells.

The ability to change the environmental pH from acidic to neutral could have important effects on the host-pathogen interaction, since many niches in the host, such as oral cavity, vagina and the skin, have acidic pH. Another environment where pH is critical is the phagolysosome, where acidic pH activates lysosomal proteases and hydrolases, potent defensive enzymes, to destroy the pathogen [32]. Therefore, we asked whether the *stp2Δ* mutant cells have a morphogenetic defect upon internalization by macrophages. First, we monitored the interaction of *stp2Δ* mutant cells with the macrophages using live cell video microscopy. As expected, the majority of wild type *C. albicans* cells initiated hyphal morphogenesis within one hour of phagocytosis (Fig. 3A; Movies S1 and S2 are the wild-type and *stp2Δ* mutant, respectively). Hypha formation continued and eventually allowed *C. albicans* to escape phagocytosis by rupturing the macrophages. In contrast, the phagocytosed *stp2Δ* mutant cells showed a severely impaired ability to form hyphae. Importantly, *stp2Δ* cells that were not phagocytosed switched to the hyphal forms, indicating that *stp2Δ* mutant cells have no inherent defect in responding to hyphal-inducing conditions in this medium. To calculate what percentage of the cells underwent hyphal morphogenesis during phagocytosis, we carefully observed the time-lapse images of phagocytosed wild type or *stp2Δ* mutant cells and counted both total phagocytosis events and morphogenesis. At one hour of co-culture about 14% of the phagocytosed wild type cells and 8% of the *stp2Δ* mutant cells had initiated hyphal morphologensis (Table 1). However, significant differences in hyphal morphogenesis between the two strains were observed at later time points: at three hours of co-culture about 75% of the wild type cells were found as germ tubes or hyphae, whereas only 31% of the *stp2Δ* mutant cells have switched to the hyphal morphology. We noted that *stp2Δ* mutant cells that had begun to germinate prior to phagocytosis continued to form hyphae within the phagosomes, but those that were ingested as yeast rarely switched morphology (data not shown). Thus, *stp2Δ* mutant cells have a substantial reduction in hyphal morphogenesis following phagocytosis.

**Table 1:** Morphology changes in phagocytosed *C. albicans* cells.

	Yeast cells	Germ tubes	Hyphae
**Wild-type**			
1 hour	85.9±3.7	12.8±2.0	1.2±1.7
2 hours	45.5±6.8	31.9±13.7	8.0±37.1
3 hours	25.1±2.1	25.9±11.8	48.6±13.9
***stp2Δ***			
1 hour	92.1±7.2	4.5±4.0	3.2±5.4
2 hours	81.3±3.0	12.2±8.2	7.16±6.1
3 hours	70.8±13.3	11.0±2.6	20.0±15.4
***stp2Δ*** ** + BafA**			
1 hour	90.4±2.0	7.8±20	1.8±1.1
2 hours	43.6±6.1	34.4±6.1	22.0±11.2
3 hours	28.4±11.7	20.4±11.2	20.4±11.2

**Figure 4:**
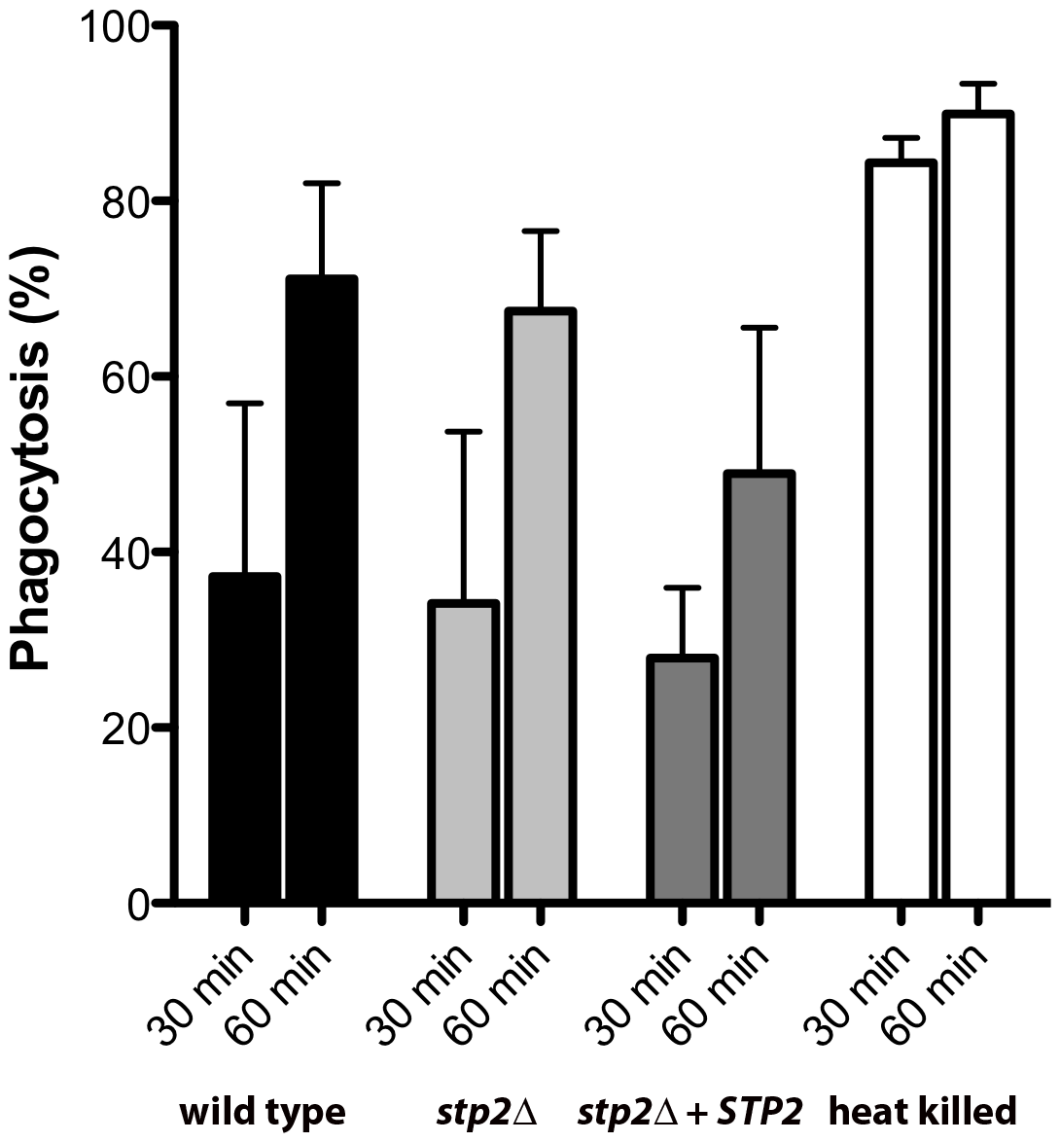
The phagocytosis rate of *stp2* mutant cells by RAW264.7 macrophages does not differ from the wild-type cells. Cells of the indicated genotypes were co-cultured with RAW264.7 macrophages for 30–60 minutes, stained with Calcofluor White, and fixed with paraformaldehyde. The percentage of phagocytosis was determined as the number of cells unstained by Calcofluor White relative to the total number of *C. albicans* cells. At least 100 cells per condition were counted.

We sought to understand whether the impaired hyphal growth might be caused by altered recognition by macrophages, as has been observed in some mutations that affect the cell wall proteome [33], [34]. We monitored the uptake of *C. albicans* cells by macrophages by applying the membrane-impermeant chitin binding dye Calcofluor White (CW) to distinguish between external versus internalized cells. We did not observe any significant differences in uptake of live *C. albicans* cells tested, indicating that recognition of *stp2Δ* mutant cells by the macrophages is not impaired (Fig. 4). At 30 min about 30% of the wild type cells, *stp2Δ* mutant cells and *STP2* complemented cells were internalized and this increased to about 60% at one hour. In contrast, phagocytosis of the heat killed cells was far more effective, with the majority of the cells internalized by 30 minutes (Fig. 4), which may be explained by alterations in the cell wall caused by the heat killing [35]. Thus, the observed morphogenesis defect in the *stp2Δ* mutants was not due to differential recognition by the RAW264.7 macrophages.

**Figure 5:**
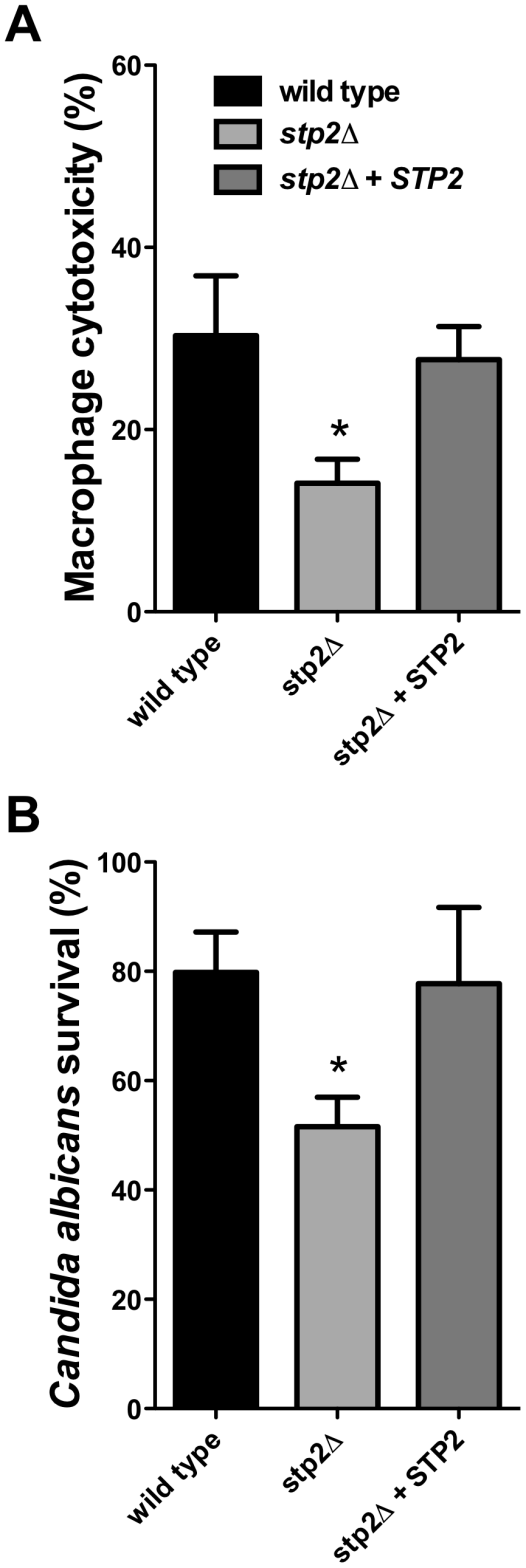
Cells lacking *STP2* show reduced survival during phagocytosis and reduced capacity to kill RAW264.7 macrophages. **A.** *C. albicans* cells of the indicated genotype were co-cultured with RAW264.7 macrophages for five hours. Cytotoxicity was determined by release of LDH, calculated as described in Materials and Methods. **B.** Killing of *C. albicans* by the immune cells was determined through an end point dilution assay in which serially dilutions of *C. albicans* cells were co-cultured with the RAW264.7 macrophages and colony formation was observed 24–48 hours after initiation of the experiment. Percent survival was calculated as described in Materials and Methods. Statistical analysis on the data was performed using a one way anova, followed by Tukey's multiple comparison; asterisks indicate p<0.05 compared to the other conditions. Results are the average of three replicate experiments.

Hyphal growth permits *C. albicans* cells to escape phagocytosis by lysing the macrophage. We hypothesized that the hyphal growth defect of the *stp2Δ* mutant strain would result in less damage to the phagocyte. For this purpose we measured loss of macrophage membrane integrity based on lactate dehydrogenase (LDH) release by the cells after five hours of co-culture. At this point about 32% of the RAW264.7 macrophages were killed (Fig. 5A); in contrast, damage caused by the *stp2Δ* mutant strain was significantly reduced (16% killed). The complemented *STP2* strain damaged macrophages to about the same extent as the wild type (Fig. 5A). This data clearly indicates that *STP2* is required for successful escape from phagocytosis.

Since we observed that the *stp2Δ* mutant has a reduced ability to form hyphae and escape phagocytosis, we asked whether it was more efficiently killed by the immune cells. To test *C. albicans* survival following phagocytosis we used an end-point dilution survival assay as previously reported [36]. Only 54% of the *stp2Δ* mutant strains survived the macrophage attack while about 80% of the wild type cells and 78% of the *STP2* complemented cells survived (Fig. 5B). Thus, the reduced ability to escape macrophages leads to more effective killing.

### Growth of *stp2Δ* mutant cells is not impaired by different stress conditions mimicking the macrophage phagosome

The impaired interaction with macrophages could results from enhanced sensitivity to host-derived stresses, which we tested using in vitro growth assays. Phagocytosed *C. albicans* cells appear to utilize non-fermentable carbon sources for nutrition rather than glucose [18]. Therefore, we tested growth in minimal medium supplemented with ethanol, acetate or glucose as the sole carbon source and found no meaningful differences (Fig. S3). Further, the *stp2Δ* mutant strain was not more sensitive to conditions that mimic other aspects of the phagolysosomal environment, including oxidative stress, nitrosative stress, or iron depleting conditions (Fig. S4). Thus, the *stp2Δ* mutants are not more sensitive to conditions found in the macrophage phagosome.

### Phagosomal neutralization promotes *C. albicans* escape from the macrophages

**Figure 6:**
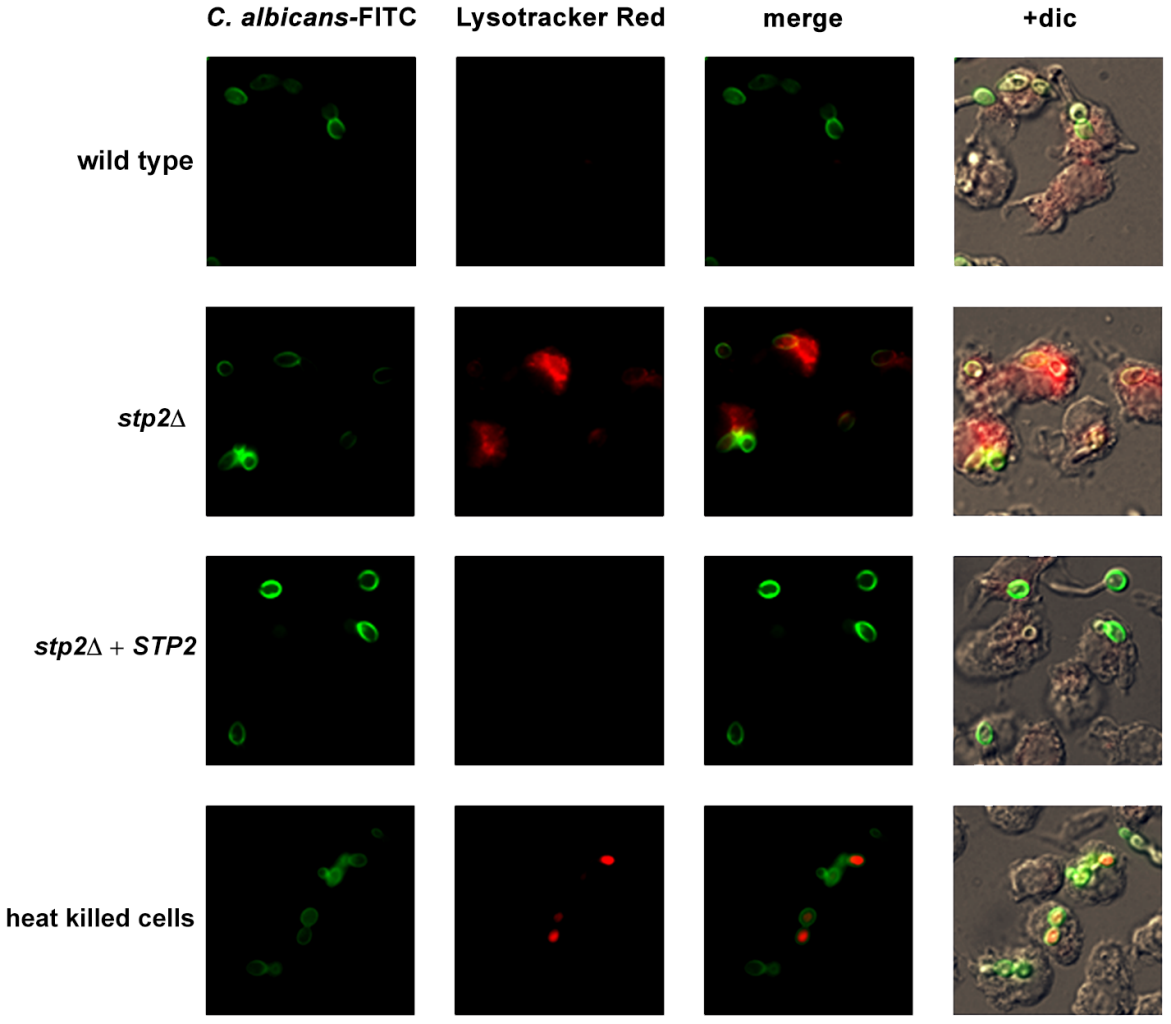
*C. albicans* alkalinizes the macrophage phagosome. FITC-stained *C. albicans* wild type, *stp2Δ* mutant, *STP2* complemented cells or heat-killed wild type cells were co-cultured with RAW264.7 macrophages preloaded with Lysotracker Red at a 1∶1 ratio and imaged after one hour of co-culture. Representative images are shown.

**Figure 7:**
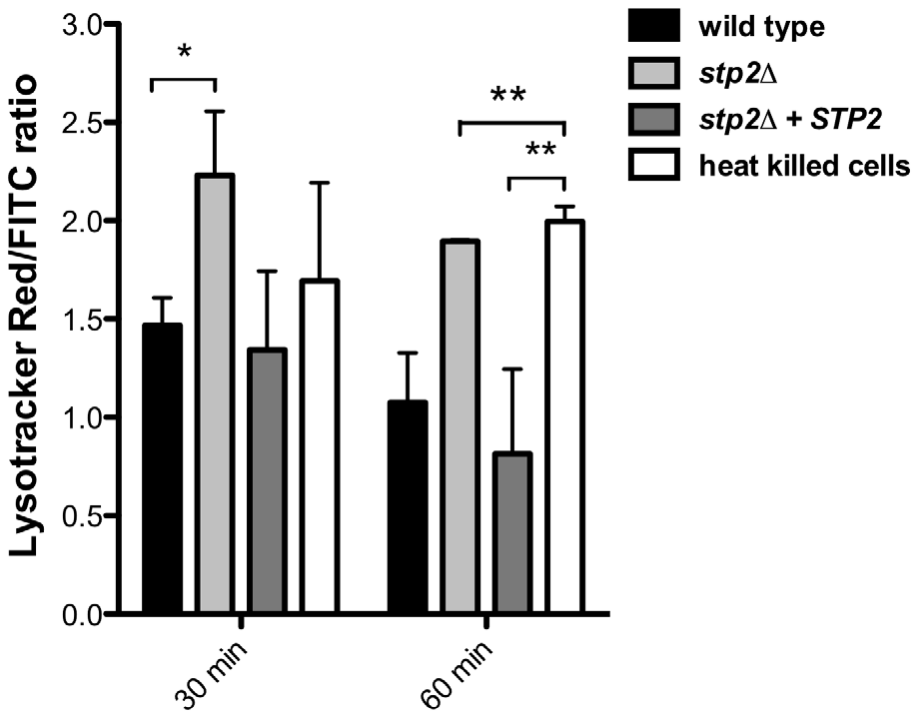
*C. albicans* cells alkalinize the macrophage phagosome. FITC-stained wild type, *stp2Δ* mutant, *STP2* complemented or heat killed wild type cells were co-cultured with macrophages preloaded with Lysotracker Red. The cultures were fixed at the indicated times, imaged and phagosomal pH was calculated as described in Materials and Methods. Statistical analysis was performed using a two-way anova; asterisks indicate p<0.05 compared to the other conditions. Data from three independent experiments is shown.

Given the Stp2p-mediated alkalinization *in vitro*, we hypothesized that the defect in hyphal growth in phagocytosed cells reflected a difference in the pH of phagosomes containing wild type versus *stp2Δ* mutant cells, *i.e.* neutral versus acidic. To test our hypothesis we pretreated RAW264.7 macrophages with the acidophilic dye Lysotracker Red DN99 (LR) to allow accumulation of the dye in acidic organelles (lysosomes). FITC-labeled *C. albicans* cells were allowed to interact with the LR-loaded macrophages for up to two hours. As expected, heat killed cells were found in acidic phagosomes one hour after co-culture (Fig. 6). In contrast, *C. albicans* wild type cells rarely localized to acidic phagosomes as noted by the diffuse LR staining, suggesting that the pH of the *C. albicans*-containing phagosome is over 5.5 and that these cells alter the nature of this compartment relative to heat-killed cells. In contract, cells lacking *STP2* localized to acidic phagosomes, similar to the heat killed cells; complementation of *STP2* restored the wild type phenotype (Fig. 6). To estimate the difference in the pH of phagosomes containing wild-type versus *stp2Δ* mutant cells, we measured the fluorescence intensity of LR relative to FITC surrounding phagocytosed cells, with the rationale that a higher ratio indicated a lower pH (Fig. 7). The LR:FITC ratio for heat killed cells in acidic phagosomes was about 2.0 at 60 min (Fig. 7). In contrast, the LR:FITC ratio for wild-type or *STP2* complemented cells was 1.2 and 0.8 respectively, while the *stp2Δ* mutant cells had an LR/FITC ratio of 1.9, close to that of heat killed cells. Stp2p-dependent phagosomal neutralization by *C. albicans* was also observed following phagocytosis by bone marrow-derived macrophages (data not shown). Thus, there is a substantial difference in the pH of phagosomes containing wild-type versus *stp2Δ* mutants, suggesting that the alkalinization process we have described *in vitro* also occurs within the phagocyte.

**Figure 8:**
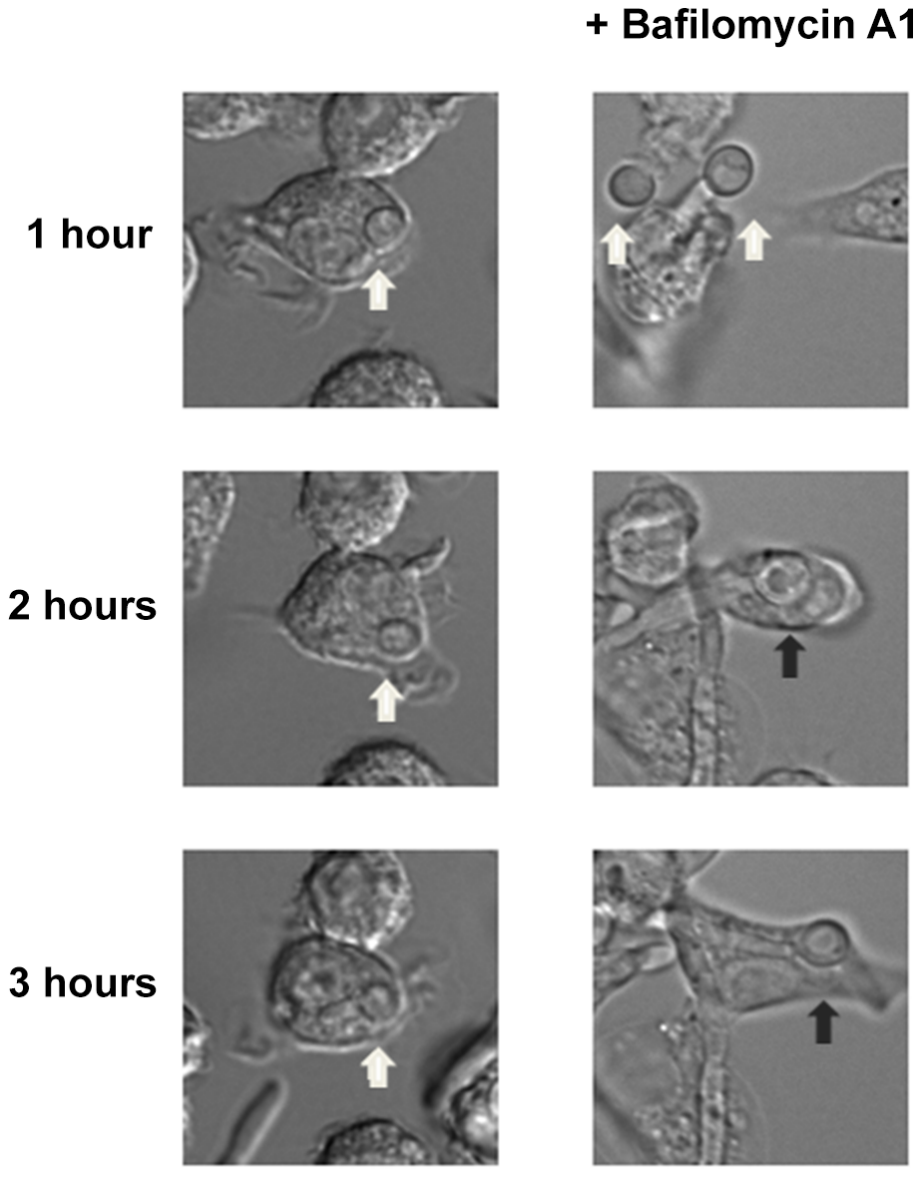
Neutralization of the phagosome is sufficient for hyphal morphogenesis and escape from the macrophages. Cells lacking *STP2* were co-cultured at 1∶1 ratio with RAW264.7 macrophages that were pre-treated with 50 nM Bafilomycin A1. Co-cultures were imaged using time-lapse microscopy. Representative images are shown, but the experiments were performed in triplicate and morphogenesis was quantitated in Table 1. White arrows point to *C. albicans* cells in yeast morphology, whereas black arrows show germinated cells.

Next, we asked if failure to neutralize the phagosomal environment alone explains the hyphal defect of *stp2Δ* cells. To test this, we created neutral phagosomes by specifically inhibiting the vacuolar ATPase (v-ATPase) of the macrophages using bafilomycin A. We verified that the phagosomes were neutralized by obtaining the LR/FITC ratio after one hour of co-culture of *C. albicans*, which was about 1.0 for all the cells tested (Fig. S5). Importantly, bafilomycin A completely suppressed the hyphal morphogenesis defects of phagocytosed *stp2Δ* mutant cells (Fig. 8). Indeed, the proportion of *stp2Δ* hyphal cells in bafilomycin-treated macrophages was essentially the same as that of the wild-type control, indicating that neutralization of the phagosome is a key factor for hyphal morphogenesis in these cells (Table 1). Bafilomycin A1 did not affect *C. albicans* growth or morphogenesis in macrophage-free RPMI medium (data not shown). Altogether, our data suggests that the observed differences in *stp2Δ* morphogenesis, macrophage survival and reduced macrophage killing are due to inability to neutralize the phagosomal environment. More importantly, neutralization of the phagosome promotes hyphal morphogenesis and phagosomal escape in the fungal pathogen.

### Environmental alkalinization can occur in body niches colonized by *C. albicans*

**Figure 9:**
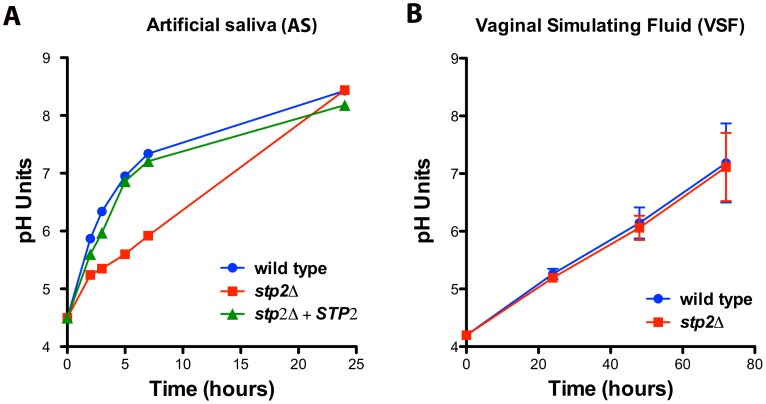
Environmental alkalinization by *C. albicans* occurs under host niche-simulating conditions. Wild-type cells, *stp2Δ* mutant or complemented cells were grown in **A.** artificial saliva, pH 4.5 or **B.** vaginal simulating fluid, pH 4.2. The pH change of the growing cultures was measured at the indicated time points. The experiment was performed in triplicate.

Since *C. albicans* can alkalinize the phagosome and this process contributes to hyphae formation and escape from the macrophages, we asked if alkalinization may alter the host-pathogen interaction in other body niches colonized by *C. albicans*. Because both the oral and vaginal cavities are acidic, we tested *C. albicans* alkalinization on media that mimics the composition of vaginal fluid (vaginal simulating fluid or VSF) and human saliva (artificial saliva or AS). Alkalinization in AS was remarkably rapid, rising from pH 4.5 to 7.0 within five hours and further to pH 8.4 at 24 hours of incubation (Fig. 9A). Similarly, these cells steadily raised the pH of the VSF from starting pH of 4.2 to 7.0 (Fig. 9B), though this was much slower, occurring over 72 hours. Interestingly, *stp2Δ* mutant cells showed a marked delay in alkalinization on AS, where the pH reached only 5.3 at five hours (Fig. 9A), but the alkalinization of VSF did not differ from that of the controls (Fig. 9B). The significant difference in the time required to fully alkalinize VS and AS, and the differential requirement of Stp2p in the alkalinization processes, suggests that multiple mechanisms for environmental alkalinization by *C. albicans* may exist.

### Stp2p is important for full virulence in a mouse model

**Figure 10:**
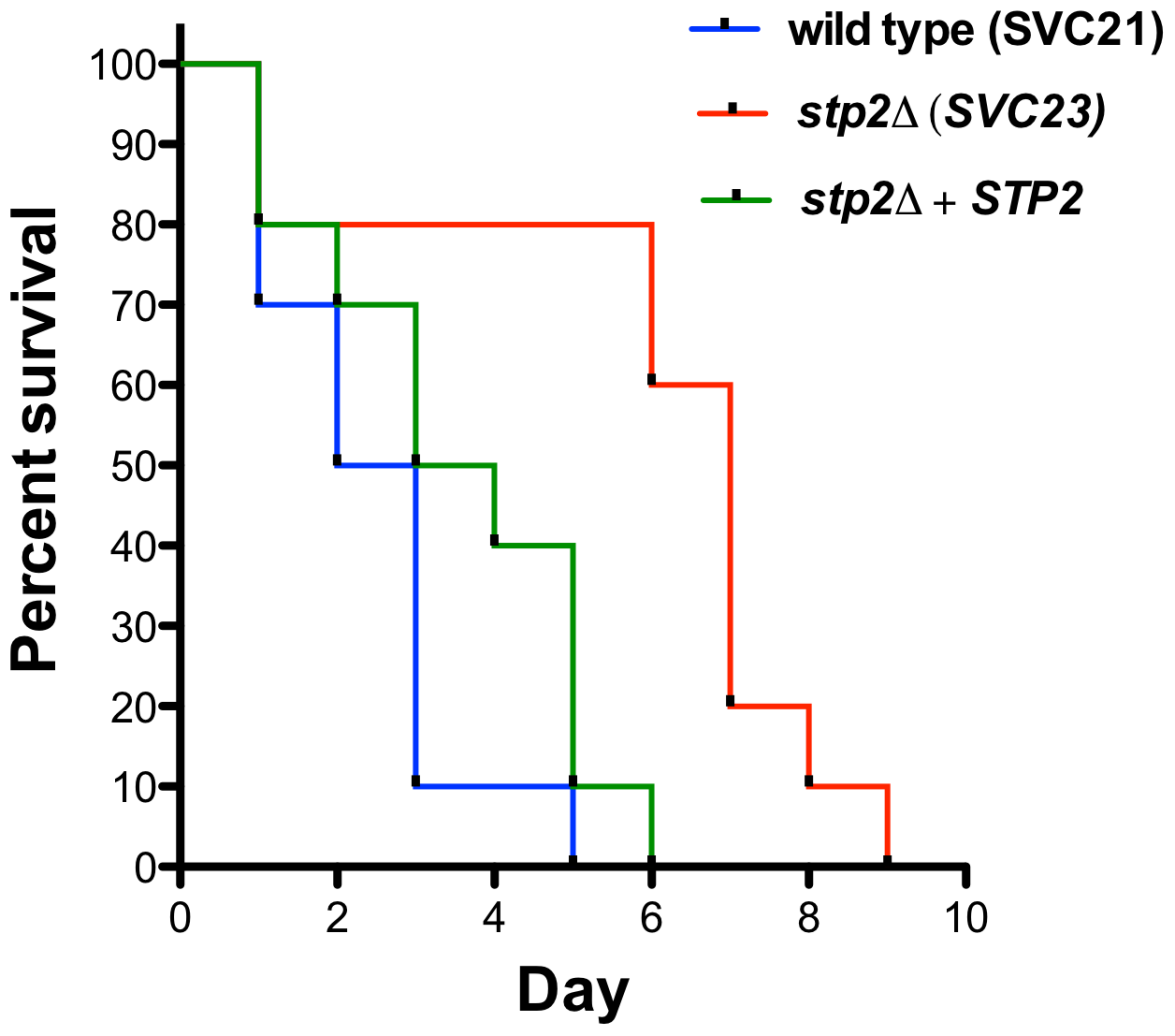
Mutation in *C. albicans STP2* causes attenuation of virulence. Outbred ICR mice were injected via the tail vein with 10^6^ cells of the wild type strain SVC21, *stp2Δ* mutant strain SVC23, or the *STP2* complemented cells resuspended in phosphate buffered saline. Ten animals per strain were used and the animals were monitored for signs of infection as described in Materials and Methods. The statistical analysis showed that the mutant survival curves are significantly different compared to the wild type or the complemented cells, with a *P* value of 0.0011 and 0.0024, respectively.

The decreased survival upon phagocytosis by the macrophages of *stp2Δ* mutants raised the question whether these strains would be impaired during disseminated infection. Therefore, we tested *stp2Δ* mutant strains in the standard mouse tail-vein injection model of disseminated hematogenous candidiasis. The *stp2Δ* mutant strain SVC23 was attenuated in virulence compared to the *STP2* complemented or the wild type strain SVC21 (Fig. 10), with a mean time to death of 2.4 days for the wild-type strain compared to 5.9 days for the mutant strain, indicating that Stp2p is required for full virulence in this animal model of infection. The statistical analysis showed that the survival curve for this mutant is significantly different compared to the wild type or the complemented cells (p = 0.0011 or 0.0024, respectively). We also tested the survival of a second, independent *stp2Δ* mutant strain SVC22, which had a mean time to death 5.3 days (data not shown). This modest attenuation is consistent with the partial reduction of hyphal induction and macrophage toxicity in phagocytosed cells.

## Discussion

Here we show that *C. albicans* can neutralize the macrophage phagosome to induce hyphal morphogenesis and escape from the immune cell and we propose the first mechanism for this phenomenon: extrusion of ammonia as a byproduct of amino acid catabolism within the host cell. This conclusion comes from the analysis of strains lacking Stp2p, a transcriptional regulator of amino acid permease genes that is required for amino acid-driven alkalinization in vitro [27]. Cells lacking Stp2p are defective in hypha formation during phagocytosis, show reduced survival upon interaction with the immune cell and are defective in killing the macrophages. These cells grow at wild type rates on alternative carbon sources and show normal resistance to a variety of stress conditions encountered in the phagosome, indicating that the observed defects are not due to an enhanced sensitivity to macrophage-derived stresses. However, *stp2Δ* mutant cells switched to the hyphal form when within neutral phagosomes, indicating a key role for pH in inducing germination of phagocytosed cells. Alkalinization is also observed in artificial saliva and vaginal simulating fluid, indicating that there may be *Candida*-induced pH changes at body sites normally occupied by *C. albicans*, such as the oropharyngeal tract and vulvovaginal tract, underlining the effect of this phenomenon on microbial and human physiology. These defects are reflected in a modest attenuation of virulence in a mouse model of disseminated candidiasis.

The intracellular fate of *C. albicans* cells is not well understood. A few studies on the phagosome-lysosome fusion following *C. albicans* ingestion by macrophages exist: two studies concluded that the fusion is inhibited, two studies concluded that fusion occurs, while another got both results depending on the assay used [37]–[41]. A key distinction in these papers was whether fusion was monitored by acidification using dyes such as acridine orange or lysotracker or by colocalization with endocytic markers such as LAMP-1. Our data provides a possible explanation for these contradictory reports, since we show that *C. albicans* can alkalinize the phagosome and that neutralization of the phagosome promotes hyphal morphogenesis and escape from the host cell. The exact fate of *C. albicans* after phagocytosis remains unclear: studies report *C. albicans* localization in mature phagolysosmes [37], late endosomes [39], or the endoplasmic reticulum [16]. Our data does not directly address this question, which clearly requires additional study, but we demonstrate a difference in the pH of the *Candida*-containing compartment between the wild-type strain and *stp2Δ* mutants, the latter of which behave similarly to killed cells. The simplest explanation is that the wild-type cells occupy a fused phagolysosome in which acidification is outcompeted by ammonia release from the fungal cell, though we cannot exclude differences in trafficking between these two strains.

We indicate here the importance phagosomal neutralization for *C. albicans* escape from the macrophages, however additional factors may also contribute to this process, including nutrient availability, CO_2_ concentration, presence of reactive oxygen species, and modulation of the iron content. The effect of these morphogenetic stimuli is likely combined, since mutants in hyphal morphogenesis, cell wall synthesis, or nutrient assimilation fail to escape from phagocytosis [16], [33]. The exact role of each of these factors on hyphal morphogenesis during phagocytosis is yet to be determined, but here we provide for the first time evidence that neutral pH is a critical component since mutants defective in environmental alkalinization remain in acidic phagosomes and do not switch to the hyphal form.

It has been well documented that pathogens manipulate the phagosomal environment to promote survival by neutralizing the phagolysosome, preventing phagosome-lysosome fusion and/or reducing the production of reactive oxygen or nitrogen species [42]–[45]. Phagosomal neutralization is also observed with *Helicobacter pylori*, which uses an extracellular urease to produce ammonia to elevate phagosomal pH and retard acquisition of late endosomal markers, allowing survival [43], [46]. Other fungal pathogens, such as *Candida glabrata*, *Histoplasma capsulatum, and Cryptococcus neoformans* alter phagosomal pH, but the mechanisms to achieve the pH increase differ from the one reported here. For instance, *C. glabrata* inhibits phagolysosomal fusion and survives phagocytosis without damaging the host cells or eliciting a proinflammatory immune response but, surprisingly, this does not require viable cells [47]. In contrast, *H. capsulatum* resides in neutralized mature phagosomes [48], [49]. The mechanism for this is unknown, but may involve permeabilization of the phagosomal membrane via the saposin-like protein Cbp1, which is secreted into the phagosomal space [50]. Similarly, *C. neoformans* appears to destabilize the phagosomal membrane such that there is exchange of luminal contents with the cytosol [51]. Thus, our findings are consistent with other fungal pathogens, though the mechanism we propose is quite different. Pharmacological inhibition of acidification also compromises macrophage antimicrobial activity: artificial elevation of the phagosomal pH using NH_4_Cl, chloroquine or Bafilomycin A1 has also been found to impair killing of phagocytosed *Staphylococcus aureus*, *Bordetella pertusis* and the fungal pathogen *Aspergillus fumigatus* [42], [44], [45]. Further, addition of NH_4_Cl to normal resident mouse peritoneal macrophages reduced the rate of killing of the non-pathogenic *S. cerevisiae* [52]. Likewise, we observe hypha formation and escape from Bafilomycin A1-treated phagosomes of the *stp2Δ* mutant cells that normally are defective in these processes. In the context of *C. albicans* the elevated pH of the phagosome will promote hyphal morphogenesis and escape from phagocytosis.

Our data suggests that this alkalinization phenomenon is driven by metabolic changes resulting from nutrient starvation imposed by the phagocyte; metabolism of non-fermentable carbon sources appears to be important during infection as disruption of some these carbon metabolic pathways impairs virulence [19], [21]–[23]. Glucose limitation within the phagosome might be one of the mechanisms macrophages use to prevent survival of the ingested cells in a manner analogous to iron limitation within the host. For example, enzymes involved in the gluconeogenesis and the glyoxylate shunt are also required for colonization and persistence of the phagosomal pathogens *Leishmania manor* and *M. tuberculosis* [53], [54]. Similarly, 3-hydroxy-methylglutaryl coenzyme A lyase, the enzyme catalyzing the last step of leucine catabolism, is important for survival of the fungal pathogen *Histoplasma capsulatum* within macrophages [55]. Amino acid utilization is important for *C. albicans* pathogenicity too, since we show that *stp2Δ* mutants have reduced survival during phagocytosis and a modest reduction in virulence. Similarly, cells lacking *DUR1,2* and *ATO5*, genes involved in detoxification and export of excess amines produced during amino acid catabolism, exhibit significant delay in hyphal morphogenesis and survival upon phagocytosis, further highlighting the importance of amino acid utilization within the host ([56]; H. Danhof and M. Lorenz, unpublished).

The source of amino acids within the macrophage is not clear, but they are a potentially plentiful nutrient source in the host. Numerous amino acid auxotrophic mutants retain full virulence in the mouse model, including those for leucine, arginine, tryptophan, and histidine [57]–[60]; this is the basis for the genetic methodology developed by Noble and Johnson [61]. This demonstrates that sufficient amino acids are accessible in the host to support growth of the pathogen, in decided contrast to the profound avirulence of nucleotide auxotrophs [29], [30], [60], [62]. We note that previous transcriptional profiling of phagocytosed cells suggested that they were starved for carbon but not for amino acids, as no amino acid biosynthetic genes were upregulated, with the exception of the arginine pathway, which we have shown is induced by oxidative stress [18], [63]. Importantly, we propose these amino acids are being used as a source of carbon and not just as the building blocks of proteins. Consistent with this, mutations of both *STP2* and *CSH3*, an ER chaperone for amino acid permeases, block alkalinization and confer virulence defects [27], [64]. The presence of extracellular amino acids activates *STP2* post-translationally to increase the relative levels of the amino acid permeases *CAN1*, *GAP1* and *GAP2*, the oligopeptide transporters *OPT1* and *OPT3*, and the secreted aspartyl protease *SAP2*; most of these genes are significantly induced in phagocytosed cells [18], [28]. Together, these indicate that amino acids are a key source of carbon, nitrogen or both within the host. Our preliminary results showing Stp2p-independent alkalinization during growth in vaginal simulating fluid indicates some redundancy with other processes; perhaps transport and utilization of oligopeptides generated from the protein in this media. In summary, we show here that catabolism of amino acids as a primary nutrient is a key determinant of the host-pathogen interaction with both phagocytes and in whole animal models. Further studies are necessary to define the most important catabolic pathways and host niches in which amino acid-driven pH changes have the biggest impact.

## Materials and Methods

### Strains and growth media

*C. albicans* strains were propagated under standard conditions in YPD medium (1% yeast extract, 2% peptone, 2% glucose). For growth on plates, 2% agar was added to the medium. To select for nourseothricin-resistant (Nou^R^) transformants, 200 µg/ml of nourseothricin (Werner Bioagents, Jena, Germany) was added to the YPD agar plates [31]. Alkalinization experiments were performed in minimal yeast nitrogen base (YNB) medium (0.17% yeast nitrogen base, 0.5% ammonium sulfate) prepared without glucose and supplemented with 1% casamino acids as the sole carbon source. For alkalinization on solid medium and for assessment of NH_3_ release by alkalinizing colonies cells were grown on GM-BCP medium, which contained 1% yeast extract, 30 mM CaCl_2_, 3% glycerol, 0.01% bromocresol purple, 2% agar and adjusted pH 4.5 [65]. Other carbon sources or chemical stressors were added to YNB medium as indicated in the results and figures. In some experiments environmental alkalinization was monitored in artificial saliva, pH 4.5 (which included, per liter, 1.7 g yeast nitrogen base without amino acids and ammonium sulfate, 5.0 g casamino acids, 1.1 g KCl, 0.5 g NaCl, 14 mg choline chloride, 10 mg sodium citrate, 1.0 mg ascorbate, 2.5 g mucin), a formulation modified from [66] or in vaginal simulating fluid, pH 4.2 (per liter, 2 g glucose, 0.16 g glycerol, 2 g lactic acid, 1 g acetic acid, 0.018 g bovine serum albumin, 0.4 g urea, 1.4 g KOH, 0.222 CaCl_2_, 3.51 g NaCl, 0.25 g mucin), a formulation modified from [67]. Utilization of different amino acids as the sole nitrogen source was assessed as previously described [28], using succinate buffered YNB medium supplemented with 2% glucose and 50 µM histidine, pH 6.0 and supplemented with the indicated amino acids at 1 mM. RAW264.7 macrophages were maintained in RPMI with glutamine and HEPES, supplemented with Penicillin/Streptomycin and 10% inactivated Fetal Bovine Serum.

### Strain construction

*C. albicans* strains lacking *STP2* were generated using the SAT-flipper method as described previously [31]. In short, approximately 300 bp of homology immediately to the 5′ or 3′ of the *STP2* ORF were amplified by PCR and cloned between the *Apa*I/*Xho*I and *Sac*I/*Sac*II sites of pSFS1. The resulting *SAT1*–FLP cassette was used to transform *C. albicans* SC5314 strain by electroporation with selection on YPD-Nou. Genomic DNA was isolated and cassette integration confirmed in the selected candidates via PCR. To eliminate the nourseothricin selection marker, the mutant strain was induced with 1% BSA in YNB medium for 4 days and the Nou^S^ colonies were selected. This process was repeated to generate the independently-derived homozygous disruptants SVC17 and SVC18 (*stp2Δ::FRT/stp2Δ::FRT*).

Complementation of the mutant strain used plasmid pSV-4, a *SAT1*-marked version of CIp10 expressing *STP2* under its native promoter. To generate this plasmid the *URA3* gene from Cip10 [68] was excised using *Bam*HI and *Sac*I and replaced by the *SAT1* gene from the plasmid pSFS1 [31] to generate pAG6. Next, pSV-4 was generated by cloning the entire *STP2* ORF into the *Apa*I and *Xho*I sites of pAG6. This plasmid was linearized with *Stu*I and used to transform SC5413 or *stp2Δ* mutant cells to generate the strains SVC21 (*STP2/STP2 RPS10/rps10:Clp10-SAT1*) and *SVC20* (*stp2Δ::FRT/stp2Δ::FRT RPS10/rps10::Clp10-STP2-SAT1*), respectively. The *stp2Δ* mutant strain was also transformed with linearized pAG6 to produce the *stp2Δ* strains SVC22 and SVC23 (*stp2Δ::FRT/stp2Δ::FRT RPS10/rps10::Clp10*). The previously generated *stp2Δ* mutant strain PMRCA57 (*stp2*Δ*4*::*dpl200-URA3/stp2*Δ*2*::*CaNAT1/stp2*Δ*5*::*MPA*) [28], was used for phenotype comparison where noted.

### Alkalinization and ammonia release assays

Alkalinization experiments were performed as previously described [27], with the following modifications: The minimal medium YNB was supplemented with 1% casamino acids as the sole carbon source and pH adjusted to 4.5. *C. albicans* cells were grown in YPD medium overnight, washed in dH_2_O and diluted to OD_600_ = 0.2 in the alkalinization medium. Cells were incubated at 37°C with aeration and growth, pH changes, and cellular morphology were assessed at the indicated times. Cellular morphology was scored by analyzing photomicrographs of at least 150 cells per condition. Experiments were performed at least in triplicate and analyzed using Prism 5.0 (GraphPad) software.

Ammonia release by *C. albicans* cells during alkalinization was assessed using acid traps as previously described [27], [65]. In brief, cells were grown in YPD medium overnight, washed in dH_2_O and resuspended at an OD_600_ of 1.0 in dH_2_O. Cells were spotted onto solid GM-BCP medium at pH 4.5; reservoirs containing 10% citric acid were placed underneath the colonies. Cells were incubated at 37°C and samples from the acid trap collected at 24, 48 or 72 hours after initiation of the experiment. Ammonia was quantified using Nessler's reagent, as described [27]. Experiments were performed in triplicate and data plotted using GraphPad Prism software.

### Growth assays

*C. albicans* strains were grown in YPD medium overnight, washed in dH_2_O and diluted in the testing medium to final OD_600_ of 0.2. For growth on alternative carbon sources we tested YNB medium supplemented with 0.01% or 0.1% acetate, 0.2% or 2% ethanol or 2% glucose. To test the effect of different stress conditions on growth, YNB medium was supplemented with 2% glucose and H_2_O_2_, DETA NONOate (Cayman Chemical), or bathophenanthroline disulfonate (BPS; Acros Organics), as indicated in the figures. Assays were performed in triplicate in 96 well plate format using a SynergyMx (Biotek) plate reader at 30°C with 1 minute agitation prior to the OD_600_ reading every 10 minutes for 16 hours.

### Live interaction with the macrophages

To assess the interaction of single *C. albicans* cells with the macrophages we seeded 3×10^6^ cells RAW264.7 macrophages to 35 mm glass bottom dishes (MatTek) and allowed them to adhere for four hours. *C. albicans* cells were grown in YPD medium overnight, diluted 1∶100 in fresh medium and grown for 3 hours at 30°C. Cells were then washed in dH_2_O and 3×10^6^ cells were resuspended in CO_2_-independent RPMI medium (HyClone) and co-cultured with the macrophages at 37°C. Images of the *Candida*-macrophage interaction were taken at 3 minute intervals for up to 10 hours using an Olympus IX81 automated inverted microscope with a temperature-controlled stage. Images from at least 10 fields were analyzed using SlideBook software. Percent hyphal morhogenesis during phagocytosis was calculated by obtaining percentage of phagocytosed cells using the following formula: (germ tubes + hyphal cells/total amount of cells)×100. Experiments were performed in triplicate.

### Macrophage cytotoxicity assay

*C. albicans* toxicity on macrophages was assessed using CytoTox96 Non-Radioactive Cytotoxicity assay (Promega) as follows: tissue culture macrophages were seeded at 2.5×10^5^ cells per well of a 96 well plate in phenol red-free RPMI and incubated overnight at 37°C, 5% CO_2._ *C. albicans* cells were grown to log phase in YPD media, washed in PBS and co-cultured with macrophages at a 3∶1 ratio for 5 hours. Calculation of lactate dehydrogenase (LDH) release by infected macrophages was then determined according to the manufacturer's protocol relative to maximum LDH release from lysed macrophages and corrected for spontaneous release of LDH by the macrophages or *C. albicans* alone. The experiment was performed in triplicate.

### End-point dilution assay

*C. albicans* survival during interaction with the RAW264.7 macrophages was assessed as previously described [36]. Briefly, macrophages were collected and resuspended in fresh RPMI medium. Cells were seeded at 2.5×10^4^ cells/well in 96 well plates and grown overnight at 37°C, 5% CO_2_. Log-phase *C. albicans* cells were washed in dH_2_O and resuspended in fresh RPMI medium. 1×10^4^ cells/well were added to wells with or without macrophages, followed by six serial 1∶5 dilutions. After 48 hours at 37°C in 5% CO_2_, microcolonies of *C. albicans* were counted using an inverted microscope in wells in which individual colonies could be distinguished. Results were presented as the ratio of (number of colonies in the presence of macrophages/number of colonies without macrophages)×100. The experiment was performed in triplicate.

### Lysotracker Red assay

For co-cultures with RAW264.7 cells, 1×10^6^ cells/ml were seeded onto glass coverslips in 12-well plates and allowed to adhere for 2 hours. Next, 1 mM Lysotracker Red DM99 (Molecular Probes) was added to the cells and incubated for 2 hours to ensure concentration of the dye in the lysosomes. In some experiments 50 nM Bafilocymin A1 (Alexis Biochemicals) was added to the cells and incubated for 30 minutes prior to co-culture. *C. albicans* cells were grown overnight in YPD, diluted 1∶100 in fresh YPD, and grown for 3 hours at 30°C. Then cells were washed in dH_2_O, stained with 1 µM FITC for 15 minutes and excess dye was removed by washing three times in PBS. Control cells were heat killed by incubation for 60 minutes at 65°C. Cells were diluted to 1×10^6^ cells/ml in phenol red-free RPMI medium and co-cultured with macrophages for the indicated time points. Cultures were washed once in PBS, stained with Calcofluor white (35 µg/ml for 1 minute) to label non-phagocytosed cells, and fixed in 2.7% paraformaldehyde for 20 minutes. To estimate the relative phagosomal pH, regions that included a phagocytosed *C. albicans* cell and including approximately 0.5 µm of the adjoining area were selected for at least 100 cells per condition. Lysotracker Red (LR) and FITC intensities were determined and corrected by subtracting background intensities in an area without phagocytosed *C. albicans* cells before calculating the LR/FITC ratio. Percentage of phagocytosed cells was calculated as (Calcofluor White stained cells/total cells)×100. Image analysis was performed using SlideBook 5.0 Image Software. All experiments were performed at least in triplicate.

### *In vivo* virulence assays

Disseminated infections with *C. albicans* were performed as previously described [23]. Briefly, *C. albicans* strains were grown to mid-log phase in YPD overnight. Next, cells were collected via centrifugation, washed, and resuspended in phosphate-buffered saline. Ten female 21–25 g ICR mice per strain were infected via tail vein injection with 1×10^6^ *C. albicans* cells in 100 µl PBS. The mice were monitored twice daily for signs of infection and euthanized when moribund. Survival data were plotted using Prism 5 (GraphPad Software) and analyzed using the log-rank test.

### Ethics statement

All animal work was performed under protocols approved by the Animal Welfare Committee of the University of Texas Health Science Center at Houston (protocol HSC-AWC-12-099). Procedures adhered to the U.S National Institutes of Health Guide for the Care and Use of Laboratory Animals, Eighth Edition.

## Supporting Information

Figure S1 **Cells lacking** ***STP2*** **show defects in utilization of amino acids as the sole source of nitrogen.** Serial dilutions of *C. albicans* cells of the indicated genotype were grown on modified YNB plates supplemented with the listed amino acids as the sole nitrogen source as described in the Materials and Methods and incubated for two days growth at 30°C. The tested control conditions should show no growth differences between the strains.
(TIF)

Figure S2 ***C. albicans stp2Δ*** **colonies are deficient in environmental alkalinization.** The indicated strains were spotted onto GM-BCP, pH 4.5. Plates were photographed after incubation for three days at 37°C. Colorimetric change of the pH indicator bromocresol purple was used as a measure for environmental neutralization as indicated by the purple halo around the alkalinizing colonies. PMRCA57 is the *stp2Δ* mutant strain generated by Martinez and Ljungdahl [28].
(TIF)

Figure S3 **Cells lacking** ***STP2*** **display normal utilization of alternative carbon sources.** *C. albicans* cells were grown on YNB medium supplemented with 2% glucose (control condition), 2% ethanol or 0.1% acetate as the sole carbon source. The pH of the media was adjusted to pH 4.5 prior to initiation of the experiment. Growth was recorded as described in Materials and Methods. The data is the average of three independent experiments.
(TIFF)

Figure S4 **Cells lacking** ***STP2*** **show normal sensitivity to macrophage-like stress conditions.** *C. albicans* cells were grown on YNB medium, pH 4.5 supplemented with H_2_O_2_, BPS or DETA NONOate at the indicated concentrations. Growth was recorded as described in Materials and Methods. Data from three independent experiments is shown.
(TIF)

Figure S5 **Bafilomycin A1 blocks acidification of the phagosome during co-culture with** ***C. albicans*** **.** FITC-stained *C. albicans* wild type, *stp2Δ* mutant, *STP2* complemented, or heat killed wild type cells were co-cultured with macrophages pre-treated with Lysotracker Red and Bafilomycin A1. The cultures were fixed one hour after co-culture, imaged and the LR/FITC ratio was calculated as described in Materials and Methods. Data from three independent experiments is shown.
(TIFF)

Movie S1 **(WT).** Wild type *C. albicans* cells phagocytosed by RAW264.7 macrophages were imaged every 3 minutes for 9 hours. Hyphal morphogenesis and burst of the macrophages can be observed few hours following co-culture.
(MOV)

Movie S2 **(** ***stp2Δ*** **).** *C. albicans stp2Δ* mutant cells phagocytosed by RAW264.7 macrophages were imaged every 3 minutes for 9 hours. The internalized fungal cells lacking *STP2* fail to switch to hyphal form and to escape phagocytosis.
(MOV)
