# Supplementary figures and images for: Modulation of Phagosomal pH by Candida albicans Promotes Hyphal Morphogenesis and Requires Stp2p, a Regulator of Amino Acid Transport
Source: PLoS Pathog. 2014 Mar 13;10(3):e1003995. doi: 10.1371/journal.ppat.1003995 (PMC3953444; doi:10.1371/journal.ppat.1003995)

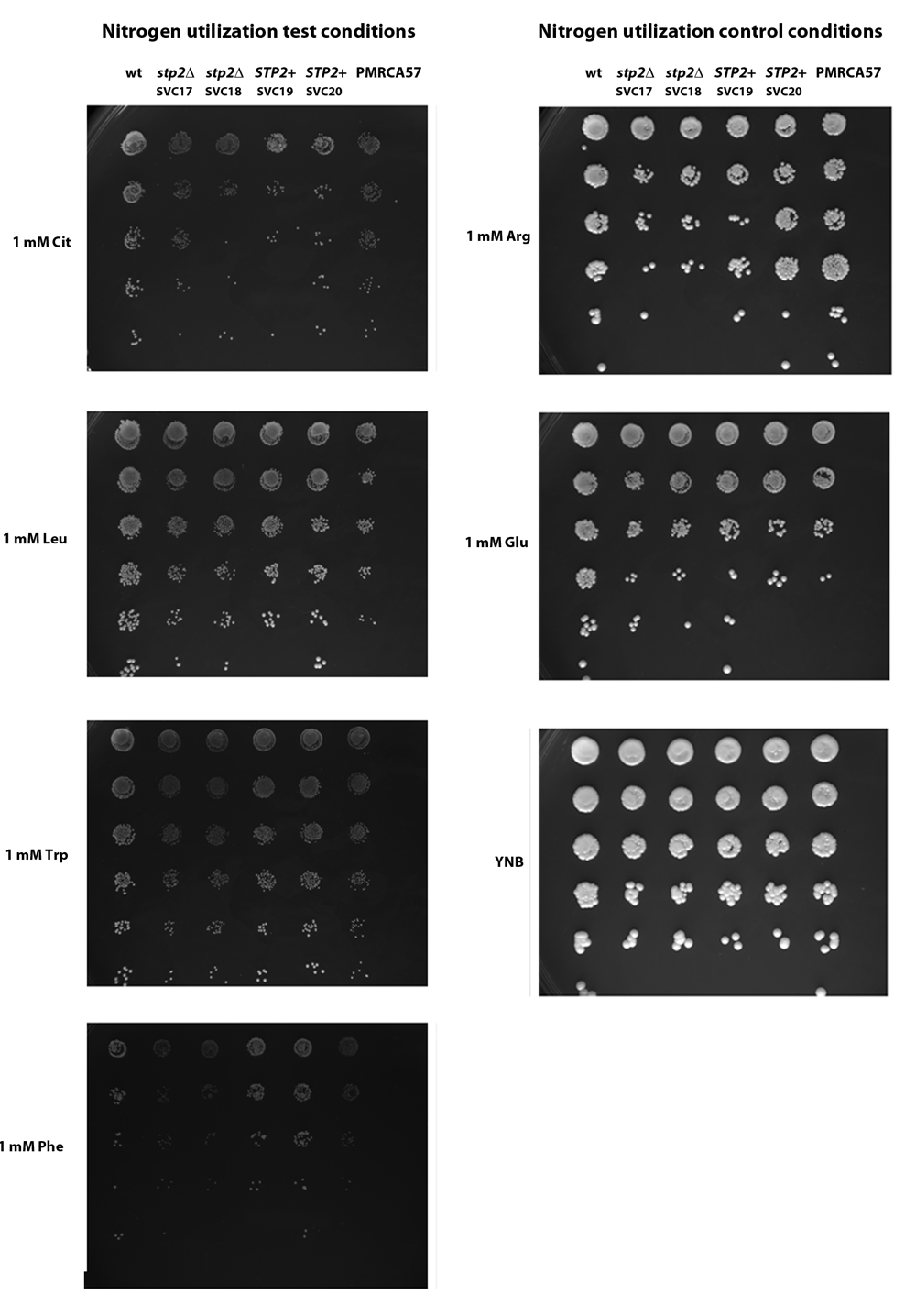

Supplement: Figure S1 — Cells lacking STP2 show defects in utilization of amino acids as the sole source of nitrogen. Serial dilutions of C. albicans cells of the indicated genotype were grown on modified YNB plates supplemented with the listed amino acids as the sole nitrogen source as described in the Materials and Methods and incubated for two days growth at 30°C. The tested control conditions should show no growth differences between the strains. (TIF) [file ppat.1003995.s001.tif]

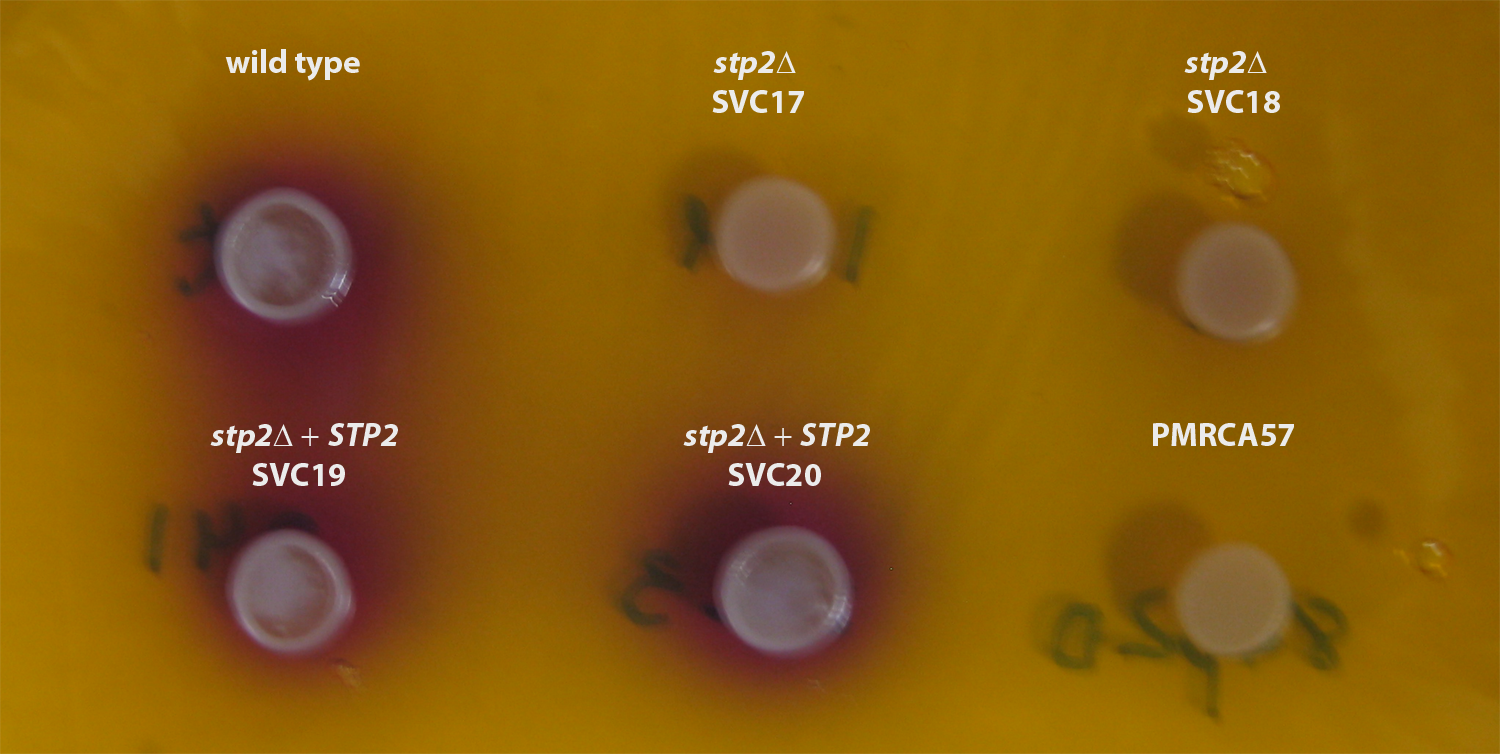

Supplement: Figure S2 — C. albicans stp2Δ colonies are deficient in environmental alkalinization. The indicated strains were spotted onto GM-BCP, pH 4.5. Plates were photographed after incubation for three days at 37°C. Colorimetric change of the pH indicator bromocresol purple was used as a measure for environmental neutralization as indicated by the purple halo around the alkalinizing colonies. PMRCA57 is the stp2Δ mutant strain generated by Martinez and Ljungdahl [28]. (TIF) [file ppat.1003995.s002.tif]

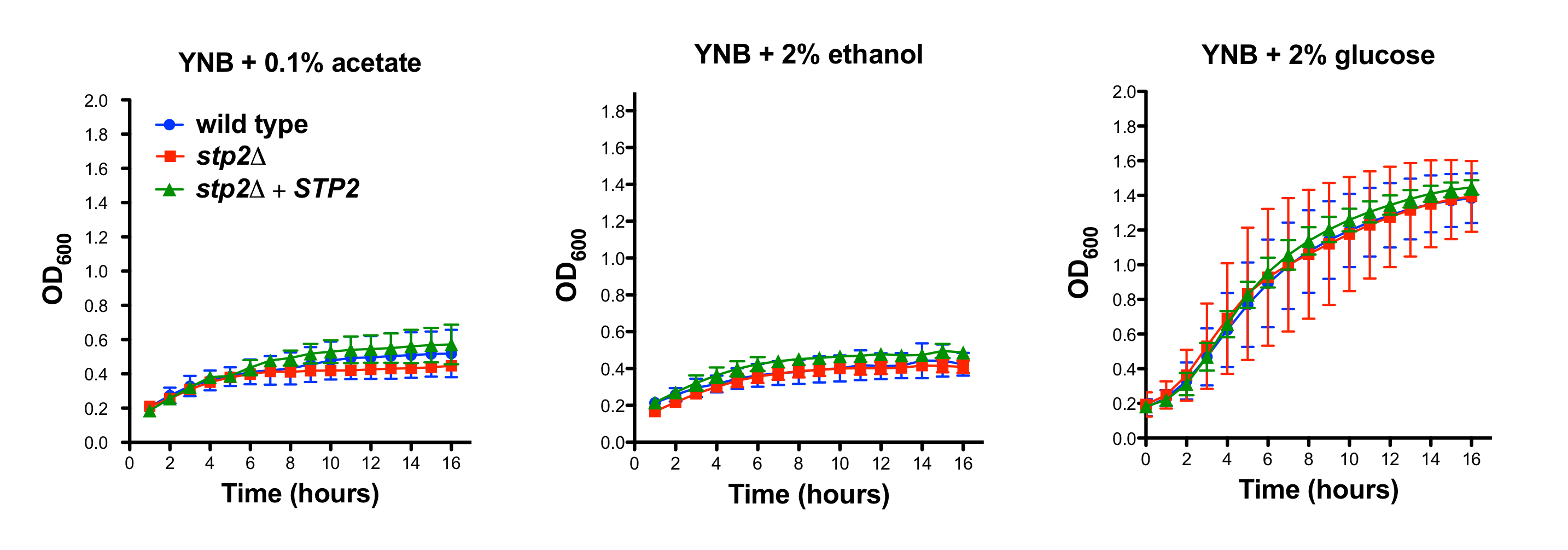

Supplement: Figure S3 — Cells lacking STP2 display normal utilization of alternative carbon sources. C. albicans cells were grown on YNB medium supplemented with 2% glucose (control condition), 2% ethanol or 0.1% acetate as the sole carbon source. The pH of the media was adjusted to pH 4.5 prior to initiation of the experiment. Growth was recorded as described in Materials and Methods. The data is the average of three independent experiments. (TIFF) [file ppat.1003995.s003.tiff]

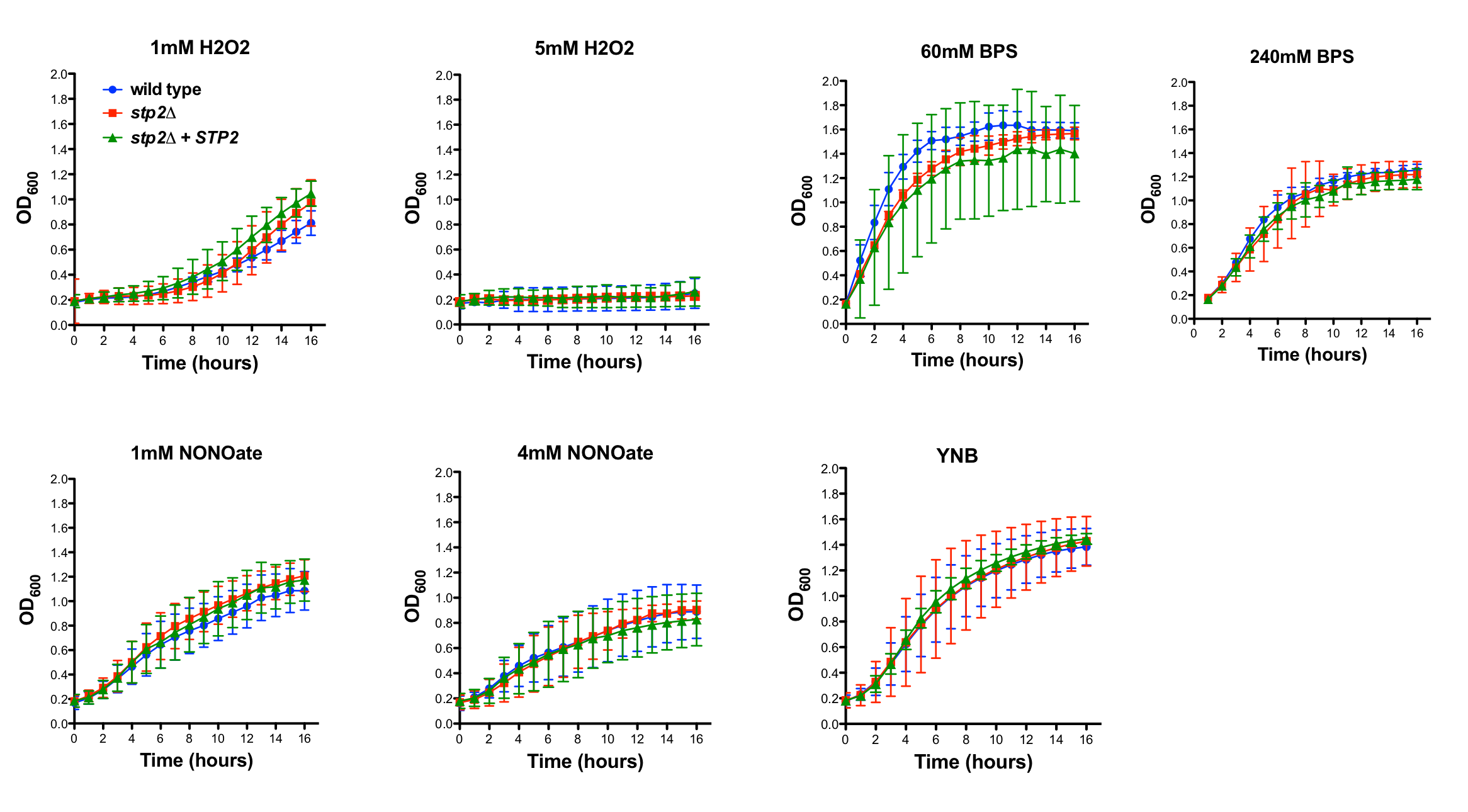

Supplement: Figure S4 — Cells lacking STP2 show normal sensitivity to macrophage-like stress conditions. C. albicans cells were grown on YNB medium, pH 4.5 supplemented with H2O2, BPS or DETA NONOate at the indicated concentrations. Growth was recorded as described in Materials and Methods. Data from three independent experiments is shown. (TIF) [file ppat.1003995.s004.tif]
